# "The Hospital" Nursing Mirror

**Published:** 1901-08-10

**Authors:** 


### "The Hospital" Nursing Mirror.

Being the Nursing Section of "The Hospital."

[Contributions for this Section of "The Hospital" should be addressed to the Editor, "The Hospital" Nursing Mirror, 28 & 29 Southampton Street Strand, London, W.C.]

### Notes on News from the Nursing World.

### THE EMPRESS FREDERICK AND THE NURSING MOVEMENT.

In the new and heavy bereavement which has overtaken the Royal Houses of England and of Germany, they will have the warmest sympathy of nurses everywhere. The death of the Empress Frederick, our own Princess Royal, which has deprived the King of a beloved sister and the German Emperor of a parent to whom he was devotedly attached, has taken away one of the best friends of the nursing movement in Germany. Following the example of her illustrious mother, Queen Victoria, the Empress Frederick not only sought every opportunity to relieve the sorrow and sufferings of others, but personally exerted herself in order to improve the conditions of nursing for the sick. So far back as 1867, when the war had revealed the insufficient supply of nursing agencies in the Fatherland, she conceived the idea of founding the Victoria House and Nursing School. Later, when the siege of Metz was over, she invited Miss Lees—now Mrs. Dacre Craven—who had volunteered for service in the war, and was in charge of the Second Fever Station of the 10th Army Corps at Marangue, to superintend her Royal Reserve Lazareth for wounded soldiers; and in 1881 she was able, after consultation with Miss Lees, to carry out the scheme. Two years later, on the occasion of the silver wedding of the Crown Prince and Princess, Berlin voted a sum of money to be placed at their disposal for the training of nurses. There was also at the same time a national offering of money to be used for the benefit of any charitable institutions they might wish to assist, and a large proportion of the two sums—exceeding a million marks—was devoted to the work of the Victoria House and Nursing School. The interest of the Empress in the organisation, which appropriately bore her mother's name, never flagged; and though she necessarily since her illness lived a retired life, she followed with attention the progress and development of nursing in the country of her adoption, as well as in that of her birth. The Empress was nursed by three sisters from Berlin, and by a nurse sent by her brother the King from England.

### THE QUEEN AND THE PENSION FUND NURSE.

It will be remembered that on July 9th a member of the Royal National Pension Fund contributed to "The Hospital" Nursing Mirror a version of the National Anthem. The author recently presented it to her Majesty Queen Alexandra, and she has been honoured by the following gracious reply:—

Marlborough House, Pall Mall, S.W.

Miss Knollys is commanded by the Queen to thank Nurse Hadley for the verses which she has been good enough to send for her Majesty's acceptance.

July 25th, 1901.

### A FRIEND OF NURSES.

The death of Lady Hilda Brodrick, who was laid to rest on Monday, has deprived the nursing movement of a very warm friend. In 1887 Lady Hilda showed her interest in it by joining the Executive Committee of the Women's Jubilee Offering to Queen Victoria, the result of which was the establishment of the Jubilee Institute. She was greatly concerned to promote the success of the Women's Memorial, and attended the various meetings of the council. She also undertook to act as president of the Surrey county organisation, and had enlisted many supporters. Nurses will share in the general regret which is felt at the loss sustained by the Secretary of State for War.

### THE WOMEN'S MEMORIAL TO QUEEN VICTORIA.

It is satisfactory to learn that the efforts of the workers in the interests of the Women's Memorial to Queen Victoria are meeting with great success amongst the people who can only afford to contribute small sums. For instance, about £3,000 has been given by about 25,000 women, including £35 11s. from Calne, which represents 888 subscribers; £7 from the Worcester High School for Girls, representing 210 subscribers; £13 14s. 5d. from the Kingston-on-Thames Girls' School, representing 285 subscribers; and £9 14s. 5d. from the branch establishments of Messrs. Lyons and Co., representing 636 subscribers. The donations of nurses amount to £150. If the movement is taken up generally in this manner, the fairest hopes of the committee, who are anxious to place Queen Victoria's Jubilee Institute for Nurses on a sure footing, may be realised.

### DISPATCH OF TWENTY NURSES TO SOUTH AFRICA.

We have received, at the instance of the Commander-in-Chief, a statement showing the nurses and dates of embarkation of 20 nursing sisters who last month proceeded to South Africa. On July 6th Miss N. Newton, of the New South Wales Nursing Service, left in the *Wakool*; on the 12th Miss C. Campbell, of the Civilian Nursing Service, and Miss A. G. Cocks, of the South Australian Nursing Service, sailed in the *Simla*; and on the 25th Miss E. Croft and Miss H. J. Henderson, of the Army Nursing Service Reserve, in the *Nubia*. On the 27th Miss M. G. Hill, of the Army Nursing Service, and the following members of the Army Nursing Service Reserve, embarked on the *Dunera*: Misses F. Baker, J. E. Bond, C. S. Carse, A. B. Denton, E. L. R. Ferguson, C. H. Harrison, A. D. Hook, D. B. Hyland, B. H. Lamb, M. Montgomery, E. Smith, A. L. Stuart, A. M. Thomson, and A. L. Von Dio.

### THE COLONIAL NURSING ASSOCIATION IN SCOTLAND.

In her capacity of president of the recently-formed Scottish branch of the Colonial Nursing Association, Lady Balfour of Burleigh has published for the information of Scotsmen and Scotswomen, and "especially for that of Scottish nurses," a letter written by Mrs. Chamberlain, which succinctly sets forth the objects of the organisation.

The wife of the Colonial Secretary repeats that the movement has the cordial support of the authorities of the Colonial Office, and pleads for further support to place the work on a sound and durable basis. Of the £5,000 required, £3,000 has already been collected and invested. There should be no difficulty in obtaining a considerable portion of the remainder from Scotland.

### THE NIGHTINGALE NURSES.

THE report of the Nightingale Fund for the year 1900, which has just been issued, shows that 39 probationer nurses, who completed their probationer year of training, were placed on the register of Nightingale nurses, and were taken on the staff of St. Thomas's Hospital. From the opening of the school in June 1860 to the end of 1900, a total of 1,645 candidates have been admitted, and 982 have, after completing a year's probationary training, been placed on the register of Nightingale nurses and received appointments in St. Thomas's or some other approved public hospital or infirmary, or district nursing institution for the benefit of the sick poor. The annual gratuity of £2 allowed by the regulations for the term of three years to the nurses who have completed a year's service satisfactorily was awarded to 94 nurses. The report states that "For candidates admitted after October 1st, 1900, these gratuities have ceased to be awarded, and it is intended that a certificate of training upon conditions to be hereafter determined will be granted." With respect to the changes that have taken place in the organisation of the nursing staff of the hospital and in the school for nurses so closely connected with it since the whole of the wards, except the two assigned to paying patients, were opened for the reception of the ordinary class of patients, the committee endorse the statement of the matron that "So far, we trust that we have kept to the principles inculcated by Miss Nightingale, and that we are entitled to feel that our nurses, when they leave us, have received such a training as she would approve." They also desire to record their sense of the value of the services rendered to the school by Miss Gordon.

### RESIGNATION OF TWO MATRONS.

THE governors of Addenbrooke's Hospital have received with the deepest regret the intimation that Miss Cureton has come to the conclusion that she can no longer discharge to her own satisfaction the arduous duties of matron, and will therefore resign her post in October. It is twenty years since Miss Cureton first became connected with Addenbrooke's, where she was trained. During the period she has held the post of matron she has enjoyed the entire confidence of the governors, and has always been greatly esteemed by the nursing staff. Miss Griffiths, matron of Lambeth Infirmary for many years, has resigned her appointment, and this resignation will also take effect in October. We regret to say that Miss Griffiths felt compelled to take this step in consequence of indifferent health. For several winters she has been laid up for some time with bronchitis, and last winter she was not able to discharge her duties as formerly. At the meeting of the Lambeth Board of Guardians last week Captain Andrew moved that the resignation be received with the very greatest regret, and Mrs. Despard, who seconded, said that Miss Griffiths had been the best officer it was possible to have. The motion was unanimously adopted, and the matter was referred to the Infirmary Committee to consider the amount of the retiring allowance which should be made.

### HOSPITALITY TO THE NURSES OF GUY'S HOSPITAL.

THE nursing staff and others of Guy's Hospital have again this year enjoyed the hospitality of the Treasurer and Mrs. Cosmo Bonsor, at Kingswood Warren. The garden parties were given on the 22nd and 23rd of last month, and the guests, numbering about 130 each day, left London Bridge, South Eastern Railway, in a special train at ten minutes to two, Mr. Bonsor travelling down with them to Kingswood, when they at once proceeded to the terrace in front of the house where their hostess was awaiting them. The weather was delightful, and after light refreshments had been partaken of, the guests wandered off at their own sweet will; some visited the church, farm, kennels, garden and stables, or explored the lovely woods and moors for heather and bracken; while others played tennis, croquet, or "ping pong," or sat under the trees listening to the Blue Hungarian band on the lawn. At five o'clock a cold collation was served in a large marquee, and at half past six the party took leave of their kind host and hostess. As the train left Kingswood, hearty cheers were given for Mr. and Mrs. Cosmo Bonsor. On the second day the guests were given permission to pick the flowers in the garden, and many of these adorned the wards at Guy's on the subsequent day.

### DEATH OF AN EMINENT AMERICAN NURSE.

WE much regret to hear of the death of Miss Emily A. M. Stoney, which occurred recently at Madison, Wisconsin. Miss Stoney was one of the best known of American nurses, and during a useful career had been connected with various leading hospitals. She was the author of several nursing text-books, and conducted the trained nursing department of that excellent periodical the *National Hospital Record* since its inception. Miss Stoney enjoyed great popularity in nursing circles on the other side of the Atlantic, and had many friends in this country.

### SHIREHAMPTON NURSING ASSOCIATION.

A VERY successful bazaar was held last month at King's Weston in aid of the Shirehampton and Avonmouth District Nursing Association. The nursing has been managed on the provident basis for the past two years, only subscribers being allowed to employ the nurse. The subscription for working people is 2s. a year, collected quarterly by district visitors. Tradespeople and villa residents pay 10s. yearly. The remainder of the fund required is raised by voluntary subscriptions, and it is hoped that in future considerable sums will be collected on board the Elder Dempster steamers running between Avonmouth and Jamaica. A collection on board a steamer making its trial trip has produced £13. For the first year and a half a single nurse worked the whole district, comprising three villages, but as more subscribers joined the number of cases became too great to be dealt with adequately by one nurse, and the committee decided to appoint a second last autumn.

### SHORT ITEMS.

WE regret that by a mistake the Helen Mary Wells Memorial Hospital was referred to as the Helen Mary Webb Hospital in our columns last week.—A libel action in the London Sheriff's Court was terminated in a very satisfactory manner on Tuesday. Mr. Dickens, K.C., who represented the plaintiff, in whose favour the decision was given, proposed that £100 should be paid by the defendant to the Queen Victoria Memorial for Nurses, and this was at once agreed to.

### Notes of Surgical Lectures to Nurses.

Delivered at the London Temperance Hospital by Dr. W. J. COLLINS, M.S., B.Sc., D.P.H.Lond., Fellow of London University and of the Royal College of Surgeons. Surgeon to the London Temperance and the Royal Eye Hospitals.

### THE SPECIAL SENSES.

A SURVEY of the animal kingdom from the lowliest to the most evolved, or a consideration of the senses in man, indicates a gradation of delicacy from so-called common sensation (in its lowest form a mere sense of externality) through touch, taste, smell, to hearing and sight. Along with this evolution of the senses goes an increasing complexity of the special organ of sense through which consciousness is affected. Of the nine pairs of cranial nerves proceeding from the brain, the first (olfactory), the second (optic), and portions of the seventh (auditory), of the eighth and the fifth (taste) are nerves of special sensation. Touch is refined common sensation, is distributed all over the skin, and acquaints us chiefly with solid bodies. Sensations of temperature, of pressure, and indeed the so-called "muscular sense" are closely related to touch. The delicacy of the sense is estimated by the smallest distance between two sensations perceived as two, *e.g.*, the points of a pair of compasses; on the tongue's tip such distance is $\frac{1}{20}$th of an inch, on the skin at the back 2 inches. Certain end organs, bulbs, or touch-corpuscles, are found in the skin and are concerned in the sensation of touch; the corpuscles are found in the papillæ devoid of blood-vessels. The sense of touch is probably conducted along the posterior columns of the spinal cord, and is often disturbed in locomotor ataxy. Section of a sensory nerve occasions anæsthesia of the parts it supplies along with certain "trophic" changes; the upper end becomes bulbous; suture of the divided ends after refreshing them may remove the anæsthesia even weeks after the accident.

Taste is refined touch; is restricted to the tongue; has relation to fluids; and discriminates between sweet, bitter, acid, and saline. The tongue is studded with fungiform and filiform papillæ, and at its base are the circumvallate papillæ. The lingual branch of the fifth nerve and branches of the glosso-pharyngeal (eighth) supply these papillæ with the sense of taste, as well as common sensation. The lingual nerve is sometimes divided to relieve pain in advanced cancer of the tongue.

Smell is restricted to the nose; is with some difficulty distinguished from taste in the case of certain flavours. It is concerned with gaseous bodies or solids suspended in a state of fine division. It is said that three-millionths of a grain of musk can be distinctly smelt. It is in the Schneiderian membrane covering the upper two spongy bones and septum that the branches of the first nerves ramify and terminate in olfactory cells.

The external ear and meatus lead to the drum-head or membrana tympani, upon which compression waves of air beat and are transmitted through the middle ear, or drum, by ossicles and the contained air (which is replenished by the Eustachian tube opening into the pharynx). In the internal ear (vestibule, semi-circular canals, cochlea) these vibrations finally impinge upon the terminations of the auditory nerve (soft portion of the seventh pair) and by the brain are transformed into auditory sensations. The range of hearing extends from vibrations as slow as thirty to those at the rate of 30,000 per second. Intensity of sound is due to amplitude of these vibrations, pitch is due to their rate; a third quality of sound is timbre. The semi-circular canals are thought to be concerned in the "sense of equilibrium," and when diseased give rise to vertigo, rotatory sensations, or forced movements. Deafness may arise from disease of the nervous apparatus or interference with the conducting media of the ear, as for instance wax or foreign bodies in the meatus, absence of air in the drum from blockade of the Eustachian tube, etc. If a tuning fork or watch placed on the head is heard better in the "deaf" ear the cause of the deafness is presumably in the conducting media rather than in the nervous apparatus, and is more amenable to treatment. Indeed it has been said with some approach to accuracy, that there are two kinds of ear disease, those that can be cured with syringing and those that cannot. The internal ear being situated in the petrous portion of the temporal bone is not very amenable to surgical interference. So-called polypi in the ear are generally granulations around necrosed bone. Perforation of the membrane is not uncommon as a sequel to the otitis of scarlet fever. Plugging the ear with cotton wool is almost always undesirable, and often very harmful. Artificial drum-heads of india-rubber are sometimes worn, and need caution in their application, removal, and cleansing.

Sight exceeds hearing in its refinement; it informs us of the fixed stars billions of miles away, and in our finest measurements the appeal is finally to sight. Vibrations in the optic nerves are set going by exceedingly rapid vibrations (at right angles to their path of progress) of ether, a substance presumed to extend throughout space and penetrate even the densest solid. The size of these ether vibrations determines brightness, the rate of them determines colour. A ray of light passing through a prism is not only refracted or bent in its path towards the base of the prism, but is dispersed, *i.e.* the white light is decomposed into a spectrum of red, orange, yellow, green, blue, indigo and violet. The red rays vibrate at the rate of 395 billion times a second, and the violet at the rate of 763 billion times; slower than the red are the heat rays, and faster than the violet are certain chemically active rays called actinic.

The "centre for vision" in the brain is located in the occipital lobe. The optic nerves (second pair) form an X-like commissure between the orbits and the brain, so that fibres coming from the right halves of the two retinæ pass by a common tract to the brain; and similarly with the left halves. This explains some cases of half-blindness (hemianopia). The whole of the third, fourth and sixth pairs of cranial nerves go to supply muscles of the eyes. The eye is a camera whereby rays of light entering through the pupil of the iris (diaphragm) are focussed (refracted) by the transparent media of the eye (cornea, crystalline lens, vitreous), into a picture on the sensitive retina which is the expansion of the optic nerve. In a normal eye parallel rays of light should be focussed accurately on the retina; if they fall short of it there is myopia or short sight; if they overshoot it there is hypermetropia or weak sight; the former is corrected by concave (minus) glasses, the latter by convex (plus) glasses. If the refraction be different in different meridians of the eye owing to unequal curvature there is astigmatism, which is corrected by cylindrical glasses. The capacity of seeing distinctly at one moment distant objects miles away, and at the next near objects as a book or a watch is owing to the power of varying the focus (accommodation) due to alteration in the convexity of the lens of the eye effected by the ciliary muscle. This power diminishes from infancy to age, and when near objects have to be held at an inconvenient distance in order to be seen presbyopia (old sight) has supervened; this can be remedied by the use of convex (plus) glasses.

All defects of vision are due either to obscurity of the media of the eye, errors of refraction, or disease of the nervous apparatus. Cataract is any opacity of the crystalline lens or its capsule; in early life, or as the result of injury, it is usually soft and is treated by needling; in senile persons it is hard, and is extracted, *i.e.*, the opaque lens is removed entire. Glaucoma is due to increased tension of the eye, is treated by eserine or iridectomy; atropine is always harmful in increased tension. Affections of the conjunctiva (ophthalmia) are best treated by not covering up the eye, and by use of an astringent lotion (chloride of zinc, gr. i or ij to ℥i). The pain of iritis is often markedly relieved by the application of a leech to the temple. Blisters are seldom useful, except in chronic diseases.

### How the Heat Cases were Treated at Roosevelt Hospital, New York.

By One of the Nurses.

During the three days that the thermometer was registering anywhere from 99° Fahr. to 108° Fahr., and the atmosphere was saturated with 72 per cent. of humidity, there was a constant demand in the Roosevelt Hospital on every possible resource for the treatment of heat cases which could be brought into play. Arrangements had been made to handle four cases simultaneously. Tons of ice were kept in readiness, the wards were crowded to the doors, every available space, even the floor, being occupied. Those whose temperature did not warrant tubbing, and whose prostration was due to alcoholism, were taken outside on the shady lawn and the hose turned on them for several minutes. Our ambulance service, which consists of electric vehicles, was greatly assisted by the Automobile Company, who kindly provided three additional coaches.

### Surgical Operations Postponed.

A special contingent of surgeons and nurses was assigned to the accident department; in fact, the entire staff was concentrated on heat cases, all surgical operations were postponed, except it was a case of life or death. Two rooms were especially fitted up with beds, bath-tubs, and small tables containing hypodermic syringes and different stimulants. An extra nurse was provided whose sole duty was to do nothing else but give hypodermic injections. As we all know in cases of sunstroke or thermic fever, the patient has an extremely high temperature, 106° Fahr.–110° Fahr., or even higher. In the worst cases the symptoms develop rapidly: the patient becomes unconscious, skin very hot, dry, and exceedingly flushed, perspiration entirely suppressed, breathing in some cases stertorous, in others barely noticeable, and a rapid, feeble, scarcely perceptible pulse.

### Reducing the Temperature.

The first thing to do is to reduce the excessive temperature. For this we adopted three different methods. First, the tub bath of three minutes, with constant stimulation by hypodermic injections of whisky, camphor and ether alternating. As, for instance, a patient came in at 12.30 P.M. with temperature 108°·6 Fahr., pulse imperceptible. He was immediately undressed and put into a bath at 60°, rubbed vigorously for three minutes, lifted out and slapped for three minutes, put back into tub for three minutes, and so on, until four baths had been given. At 12.52, temperature 104°·8; patient greatly exhausted; baths were omitted, stimulation continued; at 1.15, temperature 102°·4; 1.30, temperature 100°; 2.40, temperature 99°·6; 4.30, 104°·2; and at 5 P.M. death ensued. Another instance of a patient who came in at 6.45 P.M., with temperature 109°·8: Tubbed and stimulated as in the preceding manner; at 7 P.M. temperature was 105°·6; 8 P.M., temperature 101°·8, pulse 116, respiration 48; 3 A.M., temperature 100°·6, pulse 96, respiration 28; patient resting comfortably, pulse regular and of good force. He was discharged at the end of five days. Though this treatment was fairly satisfactory, the constant lifting in and out of the tub was extremely wearing and weakening to the patient.

### The Second Method.

We also adopted what is known as the infusion system. It differs greatly from the usual treatment by the application of ice in such cases. We put it in general use with patients whose temperature was not higher than 105° Fahr., but with no better result than by tubbing. An incision was made in a vein in the patient's arm, and about 1,500 c.c. of normal salt solution injected at a temperature of 97°. A patient was brought in July 3rd at 10.45 A.M., with temperature 104°·2; was infused by the above method at 11.05; temperature was 101°·8 Fahr., pulse 92, respiration 28.; by 6 P.M. the temperature was normal. He was discharged July 5th. In some hospitals this quick method gave great satisfaction, but our best and most beneficial results were obtained from the last method.

### The Third Method.

A tub-bath 60° was given from 15 to 25 minutes, using little friction, rubbing with ice, and keeping the patient highly stimulated. Every three to five minutes temperature was taken, when it became 102° or if the pulse became very weak the patient was lifted out, put to bed; if temperature went up to 103° he was tubbed similar to a typhoid. For example, a patient came in July 3rd at 3 P.M., temperature 109° Fahr. Tub-bath 60° was given for 20 minutes, at same time having hypodermic injections of tr. digitalis, ether, strych. sulph. and camphor; 3.20 temperature had fallen to 103°·8; 3.40 temperature 102°·2; baths were omitted and ice applied; at 4 P.M. temperature 100°·6, all medication was stopped; 6 P.M. temperature 98°, pulse 108, respiration 24. Patient weak but comfortable, slept six hours; at 9 A.M. temperature 98°·8, pulse 72, respiration 20. The patient was discharged July 5th. This last treatment proved very successful, and it was found that the patient was not nearly so much exhausted as from the three-minute tubbing.

### A Case on July 17.

A very interesting heat case was brought in Wednesday of this week at 2 P.M., temperature 109°, was put in bath at 60°, having hypodermic injections of strych. sulph. $\frac{1}{30}$ gr., camphor v. gr., and ether ♏ xxv., alternating; at 2.10, temperature 107°; 2.15, temperature 102°·6; and at 2.28, temperature 99°·6; lifted out of bath and given digitaline $\frac{1}{300}$ gr., whisky ♏ xxx.; 2.50, vomited a large quantity of greenish fluid; 2.52, temperature 96·4, pulse 100, respiration 30. Strych. sulp. $\frac{1}{30}$ gr. repeated; at 4.15 had a convulsion lasting five minutes, was given Magendie's sol. morphine ♏ v. Phlebotomy was performed, and about ℥ viii. blood withdrawn; infusion was given of salt solution at 100° Fahr. During this the patient had another convulsion, infusion was stopped; 5.10, Magendie's sol. morphine ♏ v.; 5.50, temperature 107°·2, pulse 132, respiration 30; 6, rectal irrigation, return flow discoloured; 7, temperature 105°, rubbed with ice for one hour, as the patient's condition would not allow tubbing; 8 P.M., temperature 101°·2, pulse very weak; 8.15, infused with salt sol. 100° Fahr., pulse improved slightly; 9 P.M., temperature 97°, respiration 24; 9.15, ice-cap applied to head, hypodermic injections of digitaline and strych. sulph. alternating; 11 P.M., temperature 99°; 12, temperature 99°·2, pulse 92, respiration 24, all medication omitted excepting strych. sulph. and whisky; 6 A.M., temperature 100°·2, pulse 92, respiration 24, rational and quiet, pulse regular and strong. At the time of writing the patient is improving rapidly.

### Strait-jackets Just Avoided.

During the hot wave we had 175 ambulance calls, the largest in the city; our mortality averaged 16 per cent. Twenty-four hours more of the heat and the physicians would have had to employ strait-jackets as well as ice in the treatment of patients. The old and experienced visiting physicians to the hospital were looking for this added horror. The thunderstorm of July 4th was hailed with welcome: even though temporary, it went far towards hastening the recovery of the afflicted, for doctors, nurses, everyone and everything connected with the hospital had been worn to the verge of fatigue by the extraordinary strain imposed on them; yet, strange to say, not one employé was overcome.

No Hitch Throughout the Rush.

The superintendent and the directress deserve ample credit for the orderly manner in which the work was conducted, not one hitch occurring throughout the rush. As they were responsible to physicians and surgeons for maintaining the best possible service, they necessarily had to bear the greatest burden, and the result showed their executive qualities to be of a very high order.

### Nursing at a Cottage Hospital in North Germany.

By an English Sister.

In May I was sent to a small cottage hospital belonging to our Red Cross Society, in a country village. It was most beautiful weather when I arrived, the Monday before Whitsuntide, and on reaching the station I was delighted to notice how pretty it seemed all round. We went a short cut, past a large dairy, and in less than three minutes I saw a well-kept garden with nice trees, which half hid a very pretty red brick house, over the front door of which I observed a large red cross on white stone, and knew at once that I had arrived at my destination.

The Nurse's Room.

After a kind welcome I was taken to my room, and was surprised to see how well ventilated it was, in such an out-of-the-way place. It was very lofty, and the high windows were so made that the top could be opened on the slanting system. There was also a good-sized ventilator over the door. This and the charming freshness of the apartment were most pleasing. The latter was quite plainly furnished, containing only the most necessary furniture, a bed, sofa, table, three chairs, wash-hand stand, locker, and a few hooks with a curtain over them for clothes to be hung away, and one solitary text over the bed; but yet it was so clean and bright, while a beautiful bunch of lilac on the table gave the whole a delicious scent of flowers, besides making it very cheerful. On going to the window I discovered a laburnam tree on the right, and lilac bushes in full blossom round a small but pretty grass plot close by, and opposite undulating cornfields, bordered in the distance by miles of fir tree woods or forests. After resting a little, the sister came for me and showed me over the house, also telling me my duties, which of course were very many and very varied.

The Patients.

The house was a pleasant one, and the patients on the whole very contented, many staying longer than they needed, if there was room for them and their means allowed it, which says a good deal for the hospital, as that is not a usual course. There was only one sister in charge, with a male nurse and ward-maid, and if very pressed, a woman for the day. There was, besides, a woman for night duty if necessary, who knew enough to call the sister if all was not as it should be. I suppose that in every village one finds some poor bed-ridden cripples without relations, friends, or means, dependent on the authorities, who are sent to the nearest cottage hospital if means are forthcoming, where, if their ailments allow them, they help a little in peeling potatoes, weeding the garden, sweeping it, etc. There were four such old people at my hospital, and they slept in the basement where there were four rooms with two beds each, so that they could rise, go to bed, etc., when they liked, without disturbing the real patients, and they were able to reach the garden without going down any steps.

The Wards.

The kitchen, mangling-room, and pantry were also in the basement. On the ground floor were the wards, two larger containing six and eight beds very comfortably, and two smaller ones with three and four beds, and one room for a paying patient; then the operating-room, bath-rooms, etc., and sister's room; and last, but not least, a very nice verandah for patients too ill to be taken into the garden—a great boon to patients and nurses. The wards were very simple, only furnished with necessary things, containing perhaps one picture, and no flowers as a rule, as in dear old England; but they were very clean, lofty, and bright, and the patients were always happy and comfortable.

The Nurses and Meals.

At 5.30 A.M. I found that the man and ward-maid began cleaning the wards, being ready by 6, when I came to take the temperatures, etc. At 6.30 coffee and rolls were brought round, then the patients were got ready, the operating room was prepared, all being spick and span by 9, as then I had to go in the pantry and cut bread and butter for the patients' lunch—a duty I shall never forget. I had no idea that people in bed could eat such thick slices and so much butter—I soon had several blisters, which have not yet disappeared, from bread-cutting in hospital. At 10 luncheon had to be ready, as the doctors came, mostly one after the other luckily, though it sometimes happened that they all arrived together, which is bewildering when there is only one sister who possesses two hands—how one does wish sometimes one had more! I may remark here that these Red Cross hospitals are private, hence each doctor brings his own patients. At 12 I had to superintend the dinners served for the patients, and after that I had dinner also. Then came afternoon quiet, at least it was supposed to be a resting time till 2.30, but of course that seldom happened, as often new patients arrived, small operations were performed and a thousand and one things which cropped up had to be done. At 3 coffee and rolls were brought round, and at 7 supper was served, consisting of milk soup or milk, and bread and butter. On Sundays the patients had sausage, cheese, etc., extra. Private patients very rarely came as they either stayed at home or went to the University Hospital at Bostock, a large deaconess hospital at Ludwigslust, or to our hospital at Schwerin.

The Benefactor in the Left Wing.

On entering this hospital I noticed on the left a wing which looks as if it had been added to the rest. This wing was built purposely for a lady who lives in the hospital. She manages the book-keeping, and everything outside hospital work, enabling the sister to give up more time to her patients than she otherwise could. This lady has devoted most of her life to the hospital, having seen it built after her own plans, and in spite of tremendous opposition and great difficulties. Now it is one of the most popular. But not only does Fräulein look after the books, etc.; she is also interested in the patients and sister, and if a patient needs special care, it is given not only without a murmur, but gladly and willingly. I found that all the patients loved her, she was so cheerful, and yet so sympathetic, always commanding respect, and always open to reason. I spent four very happy weeks at the hospital, in spite of scarcely having a moment to call my own, as there happened to be serious cases, and a good many patients too. At present I am doing parish work—my favourite work—in a harbour town on the Baltic.

### The Nurses of the Metropolitan Hospital.

A CHAT WITH THE MATRON.

By Our Commissioner.

Non-paying probationers were unknown at the Metropolitan Hospital three years ago. Accordingly, when the matron had shown me over the institution in Kingsland Road, and I had seen the many recent improvements, and observed that, even on one of the hottest afternoons in an exceptionally hot July, there was a delightful breeze in the wards, I asked Miss Bennett if she had seen enough of the working of the non-paying system to confirm her belief in its wisdom. She had no hesitation in replying emphatically in the affirmative, and having observed that the alterations in the conditions of nursing began when Miss Cave, her predecessor, was matron, I inquired as to those which previously prevailed.

### The Old System.

"Before Miss Cave's appointment," the matron replied, "the Committee paid a lump sum to the matron and sisters of St. John's House. The sisters belonged to a religious order, and most of the nurses were trained for three years here. Some had to finish their training after the Committee decided to change the system, but since 1899 all the nurses in the hospital have been our own."

"You still receive paying probationers?"

"Yes; the paying probationers are trained for three years, and the non-paying for four. The former have to pay a premium of 30 guineas, but they receive more than that in salary during the term. Thus, the first year the payment to them is £8, the second £12, and the third £20. Board and washing are also provided. But I found that so many eligible applicants could not afford to pay the premium that I begged the committee to make an arrangement by which those who are willing to sign an agreement to stay a fourth year are excused the fee."

### Four Years' Training.

"Then the training of the non-paying probationers is for four years?"

"That is so; and in signing an agreement they undertake to serve for the third and fourth years on the private nursing or the general staff of the hospital as the matron may require. The salary is raised the fourth year to £26 if the nurse is attached to the hospital staff, and to £28 if she is engaged in private nursing. I have two whose work is entirely is private nursing, and I expect to increase the private staff considerably as time goes on. The paying probationers serve in their third year on the private nursing staff, if I require."

"Do many of the nurses stay on at the hospital after they have finished their training?"

"Occasionally. One of our present sisters was trained here. But, I suppose because the training is thought to be good in the nursing world, many of the Metropolitan nurses have been most fortunate in getting appointments. One is sister at the East London Hospital for Children at Shadwell, and another is sister at Dulwich Infirmary. Two belong to the Army Nursing Service Reserve, and went out as Princess of Wales's Nurses to South Africa in July last year. They are now in Natal, and I hope soon to have them back."

"You have kept their posts open?"

"I said I would keep the wards open for them for six months, and engaged sisters in place of them temporarily; but I have extended the time owing to the situation in South Africa and the need of good nurses. They have had heavy work with the enteric patients. Sometimes they have had men walk into the wards in the first or second week of the fever."

### The Importance of Character.

"Now about the details of the training here. Do you find it difficult to secure suitable probationers?"

"It is easier to get a good nurse than it is to get a good woman. I attach the utmost importance to character, for I feel that a good woman will always do what is right, whatever her occupation. That is why I see no value in State registration. You cannot register a character."

"What is the course of instruction?"

"During their first and second years lectures on nursing and bandaging are given by the matron, on surgical nursing and anatomy by one of the visiting surgeons, and on medical nursing and physiology by one of the visiting physicians; examinations both written and *viva voce* are held as well as a practical one. The habits of the nurses are I think admirably tested by the latter."

### Tests of Habits.

"Will you instance some of the things that they are set to do?"

"They are told to wash their hands, and some of them on the last occasion lost marks because they forgot to take off their cuffs, while others omitted to clean their nails. Again, when they were required to change typhoid beds some did not scrub their hands after taking off a soiled sheet, but only carbolised them. In the third year they have lectures on gynæcological nursing, and a final practical examination in every subject before they leave."

"How many nurses are there on the staff now?"

"Thirty-three probationers in different stages of training, and eight sisters, including the night sister and the house sister. The staff has been increased by nine since the opening of the new wards on July 1st. There are now a hundred beds, and an average ward contains 20."

"What is the number of nurses allotted to each ward?"

"Four, and a sister during the day; two nurses at night, the night sister being in charge of the whole of the wards. In the children's ward of 16 beds there are two staff nurses on duty in the day and two at night. We call them staff nurses when they are in their third year. Sometimes we have probationers who have previously had one or two years' training in other hospitals."

### Hours and Holidays.

"When do the staff nurses and probationers come on duty in the wards?"

"At 7, and they leave off at 8.30. Half an hour is allowed for dressing and lunch, and the same for dinner, tea, and supper. The off-duty time is two consecutive hours before 8 P.M., and one hour from 9 to 10 P.M., so that the staff nurses and probationers are four hours out of the wards. They are off duty one half-day a week from 7 A.M. to 2 P.M., when they have breakfast in bed; or from 2 to 10 P.M., with late leave when asked for; also a whole day a month. On Sundays they are off duty from 10 A.M. to 1 P.M., 2 to 6 P.M., or 6 to 10 P.M., giving them time to go to church. Prayers are said in the hospital chapel every morning and evening, and all the nurses and maids attend."

"And holidays?"

"Fifteen days at the end of the first year, and on the completion of their second year probationers to have a month's holiday, which some of them take, by permission, in instalments. The sisters have a month's holiday each."

"What are their hours?"

"From 8 A.M. to 9 P.M. They have half an hour for breakfast, an hour for dinner, half an hour for tea—which is taken in their room—and half an hour for supper. The sisters get two hours off every day, half a day a week, every other whole Sunday, with half Saturday."

THE ACCOMMODATION FOR NURSES.

"I notice that the sisters have a bed-sitting room off the wards?"

"Yes; and they prefer their small but convenient rooms adjoining the wards, because they can be off duty, yet in call."

"The probationers have a sitting-room to themselves?"

"With the staff nurses. There is a piano, and there is also a library which is much appreciated. Several books have been given, but we have purchased some of them. The subscription is 1s. a quarter, and fresh books can be had twice a week. There is a medical library as well, from which books can be borrowed."

"Do the nurses all have meals together?"

"Yes; the sisters and myself dine with one detachment, and the home sister with another. Great care is taken with respect to the food. The committee do not stint us in anything, and are most anxious that the table should be liberally supplied."

A DREAM OF THE FUTURE.

"But you still suffer the disadvantages of cubicles?"

"Unfortunately, the sleeping accommodation we need is only a dream of the future. The cubicles are as nice and as airy as we can make them, but they ought to be rooms."

"Do all the nurses sleep in them?"

"All but four, who have to sleep out."

"I suppose that your full dream is a nursing home?"

"Yes, when we can get the money. We have plenty of ground, both to extend the hospital and to build the home; but we are very poor. If it had not been for the Zunz bequest we could not have added the new wards, and but for the liberal grant from the Prince of Wales's Hospital Fund we could not keep things going."

THE VALUE OF THE TRAINING.

"Coming from the London Hospital yourself, what do you think of the value of the training here as compared with that given at larger hospitals?"

"My object has been to go as far as possible on the lines of the London. I was trained there, and was afterwards sister of the gynæcological and obstetric wards for three years. I think that the nurses get an unusually good training here. There is a very large out-patient department, and every nurse has the advantage of three months' training in the operating theatre. For two years we placed thirty-six beds at the disposal of the Metropolitan Asylums Board for typhoid cases, and although we have necessarily ceased to do this, the training is still very varied and complete. I have never seen asepsis better taught. The doctors are most particular, and seeing that ways and means have always to be taken into consideration, everything is remarkably good."

"You have no garden?"

"Only the little strip in front. But people are very kind in giving us theatre tickets, and the Palace Steamer Company lately sent us twelve saloon passes to Ramsgate. There is no drawback to wearing outdoor uniform in the East End as there is in the West End. In fact, I prefer it, but it is not compulsory. I should like to add that no body of nurses could be more loyal to their *alma mater* than those of the Metropolitan. They have beautified the Mortuary Chapel, and they are always buying little things to adorn the wards."

### Nurses' Meals in Hospital.

THE appointment of lady housekeepers in our hospitals to relieve the lady matron of all household duties, has undoubtedly caused great reforms to be made in the commissariat of those institutions. Anyhow we do not hear so many grumbles about the food which is served or not served for the nurses.

I know from experience the difficulties of catering for a large number. One easily gets into a groove and finds it work well perhaps, from the housekeeper's and cook's point of view, but oh, the monotony of sitting down (perhaps with little appetite) to the same joint and pudding on the same day in each week, month in and month out.

Of course the bills have to be considered, and no extra expense must be incurred; it is often this fear which prevents any change being made. But variety must be obtained somehow, and this can easiest be done by giving one table or so many nurses in turn the special dish or dishes once or twice a week; this can be done with very little trouble, using up cold meat, or something which is in season and plentiful—the same with regard to vegetables and sweets.

Take peas for instance; there is neither time for shelling nor money for buying them for every table on one given day, but there is nothing to prevent sufficient being cooked for each table in turn.

A little dinner after the style of the following menu given weekly to each table would, I feel sure, be much appreciated by the partakers, as breaking the routine of joints, roast and boiled, and yet not really costing any more money. I give a suggestive menu for one day with some of the recipes necessary, and hope to give some others another time.

*Menu.*
Fillets of fish in batter.
Miroton of beef.
Creamed potatoes. Braised carrots.
Chocolate blanc-mange.

*Fillets of Fish in Batter.*—Take for these plaice or fresh haddock, wash and dry the fillets and cut them into strips about an inch wide and three long. Make a batter by mixing together four ounces of flour, a tablespoonful of salad oil, pinch of salt, yolks of two raw eggs, a quarter of a pint of cold water, and lastly the whites of the eggs whipped stiffly with a pinch of salt. Dip the fish into this batter, and fry in clean boiling fat for several minutes. Take up on kitchen paper, and keep quite hot till all are done.

*Chocolate Blanc-mange.*—Boil a pint and a half of milk. Mix in a large basin two and a half tablespoonfuls of cornflour, one tablespoonful of Fry's cocoa, and two ounces of sugar with a little cold milk. Pour the boiled milk on to these, and stir till smooth. Then return all to the saucepan and stir till it boils. Simmer for five minutes, then pour the mixture into a mould that has been dipped into cold water and set aside to get quite cold before turning the blanc-mange out into a glass dish. It should be made the day before using if possible.

### To Nurses.

WE invite contributions from any of our readers, and shall be glad to pay for "Notes on News from the Nursing World," or for articles describing nursing experiences, or dealing with any nursing question from an original point of view. The minimum payment for contributions is 5s., but we welcome interesting contributions of a column, or a page, in length. It may be added that notices of entertainments, presentations, and deaths are not paid for, but, of course, we are always glad to receive them. All rejected manuscripts are returned in due course, and all payments for manuscripts used are made as early as possible after the beginning of each quarter.

### Beyond the Seas.

### NURSING AT WINNIPEG GENERAL HOSPITAL.

(*Concluded from page* 236.)

At this time the final examination for the senior class took place and caused much excitement amongst our own class, as we felt that only one year now stood between us and our own fateful day. The lectures and papers which had been given during the winter were eagerly read up again and the lives of the house surgeons were made miserable for a short time with incessant demands for lectures and any hints which might be of value to us. I fear that this enthusiasm did not last very long, for when the summer came we found our spare time more profitably spent in bicycling, tennis, and other outdoor amusements.

### Two Frights.

During my night duty, how frightened I sometimes used to be. I remember one night hearing unearthly sounds in the basement, and not knowing what they were I thought it my duty to investigate. So armed with a large stick I went downstairs and made my way into what I thought was the empty kitchen. Imagine my terror on beholding a strange man groping wildly in the dark with his arms. I grasped my stick more firmly and prepared to grapple with what I imagined to be a violent lunatic, when just at that minute the man found the electric light which hung just above his head, turned it on, and I recognised the new night engineer who had come over from the general wards to see if the furnace was running properly. I retired very humbly with my stick hidden under my apron, and wrath in my heart—for I hate to be frightened out of my wits more than anything else. After that episode I was more prepared for "events," and felt only moderately alarmed when one night the door of one of the sections opened and a patient rushed into the kitchen where I was sitting copying out a chart, with the alarming news that Smith and Brown were killing one another in Ward I. I walked as calmly as I could into the section and closed the door so that my other wards should not be disturbed, and then I wanted to laugh badly, for the fun of the thing struck me. All the patients in the ward were able to be up, and they came flying out simply terrified. When at last I managed to look in there were my two brave men thumping each other at one end of the ward. I was horribly alarmed, but I thought it would not be professional to show it, so I walked boldly in, and taking one man—who was about 5 feet—by the collar, and the other—who simply towered above me—by the arm, I managed to get them apart and marched them down the corridor to their own ward, accompanying the triumphal procession with a flow of eloquence which I thought at the time, and they evidently thought so too, was positively a marvel. My night duty lasted for a little over three months, and I was then transferred to the general wards again and worked between the different men's and women's surgical and medical wards for some months, receiving a particularly good training in typhoid, which was prevalent at that time in the city.

### The Final Examinations.

The following Christmas I again spent on the private-ward flat, but this time in charge. My next move was to one of the large wards again as second nurse, and there I stayed until my turn came for my training in the operating theatre. Surgical work I always found very interesting, but it never had for me the same fascination as ward work. Whilst I was in the theatre the final examinations for our class were held, and there was great excitement in the whole school, but especially in the class that was most interested. I never studied very much before, but when I realised that only one short week stood between me and those final papers I read for my life. With the aid of one small candle I pored over "Clara Weeks" and "Isabel Hampton" long after the lights had gone out, and when at last I was simply too sleepy to read any more I put both books under my pillow hoping that I might perhaps dream their valuable information into my head. At last the day arrived, and we all sat down to write on the first paper. I think that day it was anatomy and physiology, but whatever the subject, I know I felt that I was hopelessly plucked when I first gazed on those type-written questions. The next day the paper had one or two "emergency" questions, and they happened to be just the things that we had not looked at for a year or two at least—the simple kind of question that was least expected, in fact. One was, "How would you treat asphyxia from drowning?" and when the paper was finished, and we gathered together to discuss it, we all felt anxious to know what the others had done for the unfortunate drowned man. "What did you do with him?" said one. "Oh, I turned him face downwards and shook the water out, and used Marshall Hall's method, and all sorts of things," said the second. "Didn't you take the sea-weed out of his mouth?" said the third. "No, I never thought of that; what on earth would he have sea-weed in his mouth for?" "Oh, they always do. I don't know why, only I'm sure it says so in 'Clara Weeks', etc."

### Graduation Day.

Then there was the treatment of frost bite, another catchy question, but one which we were able to answer easily, as we had all had practical experience in nursing it. In fact we were so much in it, that one nurse carried her treatment as far as amputation, which was certainly a bold step for a nurse, who must "never diagnose and never prescribe," to make. We did not hear the results of the examinations for a week after, and it was a happy ending to our suspense when the superintendent told us that we had all passed and were to receive our medals and diplomas from the President of the Board of Directors in a week or two. Graduation day arrived, and after the presentations, speeches were made in which we were referred to as a most wonderful class of nurses, and of course we believed all the pretty things that were said—as they were addressed to us. One of the doctors said that our only fault was that we drank too much tea; of course we were indignant, and two nurses made a resolution to give up tea and coffee for a week, just to show that they could if they wanted to do so. I was one of them, and can speak from experience of how we suffered. I was working in the theatre at the time, and it happened to be the week that the Annual Exhibition was held in town. This always meant accidents coming in and a very busy time generally, but especially so for the theatre nurses. We were often up late at night, and when at last the work was done and the theatre cleaned up and tidy we used to go down to the ward kitchen, where a sympathetic night supervisor would have a dainty little meal for us, with an inviting cup of tea or coffee to raise our drooping spirits. That week, of course, I had to refuse and content myself with cold milk or water. We both had, or imagined we had, headaches every day, and felt completely miserable; so that when the week was over and we had proved how strong-minded we were, we invited quite a party to help us partake of our first cup of tea—and certainly never before or since did a simple cup of tea taste quite so delicious.

### In Charge of a Ward.

After my theatre and maternity training were over I was put in charge of the men's ward. As this was the same ward that I had started in as a probationer three years before, it was very pleasant to me to take charge of it, and in my turn to train probationers. I never forgot to tell them, as I had been told, that they were to "always remember they were my nurses and to do me credit wherever they went." Six months after I graduated I decided to leave the hospital, but it was with a very heavy heart that I bade good-bye to the nurses, wards, home and gardens of the place where I had spent the three happiest years of my life.

### Lectures to Head Sisters.

By E. Margaret Fox, Matron of Tottenham Hospital.

LECTURE II. (*Continued*).—ON WARD MANAGEMENT

A rough division of the work in a ward of ten or twelve beds, where three nurses are allowed on day and one on night duty, would be something like this: The head sister should have special charge of five or six of them, choosing two or three of the worst cases, the senior probationer should take four or three, and the junior two, these last being preferably convalescents, as her work and want of experience unfit her for the charge of very bad cases

In a children's ward, the head sister should attend to the tiny babies herself, as they need more care than older children.

Having apportioned the patients, the head sister should, as far as possible, make the nurse in charge thoroughly responsible for them. She should wash and feed them attend their dressings, mark their charts, learn all she can about them; only—and this is very important—the head sister must always see that the work is done right.

Having arranged how many patients to give to each nurse, the head sister should think out her own work first, taking care not to absorb too many of the manual routine duties herself, or she will find she cannot get through them. She must remember that a good deal of her time is likely to be taken up in going round with the doctors, or in attending operations, and if, while she is doing this, she is worrying because she has not performed some routine duty, she cannot have an open mind to receive and remember any orders the doctors may give. Besides this, it is not good management to be always asking others to do your work for you, which you will have to do, if you set yourselves too many routine tasks. Such things as sweeping and dusting, washing up, scrubbing mackintoshes, cleaning lamps or inkstands and washing bandages, ought not to be considered as part of your regular work, although, of course, you must do them if alone on duty, short-handed, or extra busy. But you must always hear the night report in the morning and yourself write it at night. When off duty in the evening, write it as far as you can, before you go, leaving empty spaces where further directions are likely to be given, and give your senior probationer full instructions about it. Also, go over the written report with the night superintendent, adding any verbal directions needed.

You should also write the daily diet lists yourself, the orders for stores and dressings, and daily revise the medicine list. And it will be better too, for you to have personal charge of linen, medicine, and store cupboards, and to give medicines and stimulants yourself, when on duty.

The senior probationer will have to be responsible for your work when you are off duty, and for the junior's when she is away. She should have charge of test-table, dressing-waggon, doctor's-table, half the lockers and cupboards, and on alternate weeks with the junior probationer, be responsible for the order of the ward-kitchen, bath-room, and lavatory.

The junior probationer should sweep and dust the ward, wash the lavatory utensils, window-sills, bed-tables and lockers, unless some of these duties are done by a ward-maid. She should also, when on duty, give bed-pans, scrub mackintoshes, attend to the fire, wash up, clear away dirty dressings, polish bright things, and get soiled linen ready for the laundry. It is not fair to expect a probationer to clean all the dirty heads in a ward, nor to wash the feet of all the patients. Each nurse, as she washes her own cases, should finish doing them entirely, and not leave part for someone else to do. The junior should be responsible for the cleanly condition of the heads, feet, and back of her own two patients, and she may, of course, help with others that cannot be done by one alone, but that is all. Both probationers should take turns in being with you when the house surgeon makes his rounds. Never allow them both to disappear, one into the kitchen, the other into the lavatory, and leave you to go round alone. It looks slack and unprofessional, and is inconvenient if something needs to be fetched.

Having thus sorted out the work, write the rules for each probationer on paper, together with a time table of her duties, read them over to her, and be sure she understands just what you expect. This will prevent things from being forgotten or left, because they are nobody's work. Give a fresh copy to each new probationer as she comes to you, with any alterations you feel are needed. This method will keep you thoroughly well posted in the work of the ward, and you will know at a glance what each one should do and be responsible for. Besides, though you cannot always adhere to your time table with mechanical precision, it is a great help towards punctuality, and an immense factor in encouraging smartness.

As an aid in drawing up your time tables, it may be helpful to consider how much attention the various items in a ward need to keep them in order. Of course, your wards are "spring-cleaned" in due course, and then you are installed in them to keep them as they should be kept. With window cleaning and chimney sweeping, you doubtless have not much to do, as that is probably arranged for you, but it will fall to you to tell the night nurse to let the fire out about 4 A.M., to remove from the ward anything that may be spoiled by the soot, and to provide dust sheets to protect your beds, &c. The evening before the sweep comes is also a good time for taking down and washing pictures and for having the walls well swept, and in sweeping the walls, see that those in the lavatory, bathroom, and kitchen are not forgotten.

With regard to ward fires. They need a good deal of attention nearly all the year round, and in this changeable climate it is always well to have the grate laid ready for lighting, even in summer. When it is necessary to have them night and day, do, however small they may be, keep them bright. A bright fire and tidy hearth adds so greatly to the pleasant and comfortable appearance of your ward, whereas, if there is only dull blackness to be seen, choked with ashes, and an untidy hearth covered with bits of orange peel, matches, and paper, it looks both unprofessional and most comfortless. Keep a hearthbrush hanging on a nail by the side of the fireplace, and make your junior probationer responsible for keeping it tidy. Do not allow her to go off duty without first sweeping it up. Have the ashes taken away in a dustpan three times a day, and do not let the patients poke the fires or add fresh coals. A great deal of waste is caused in this way. And see that in a children's ward the fire is always well guarded. Do not air any articles on the guard or fender, where there is a possibility of their catching fire, but hang them at a safe distance on the back of a chair. Wet things, of course, must not dry in the ward. They cause an unpleasant smell, and make the air damp and most unwholesome. Have your fires made up at regular times. They should be bright and nice when the day staff come on duty, so that they will last till about ten. Then they want making up so that they will last till after the patients' dinner time, and not require attention during the doctor's visit, or while dressings are going on. Make them up again before the midday sweeping and dusting, so they need no replenishing during the rounds of the visiting staff, again after the patients' tea, then just before they settle off to sleep, so that they shall not be aroused by a mighty noise, and always before you go off duty for the day, so that the night nurse sees a cheery blaze to welcome her.

(*To be continued*)

### Echoes from the Outside World.

### AN OPEN LETTER TO A HOSPITAL NURSE.

THE death of the Empress Frederick overshadows all other events of the week. With the universal sorrow which it has provoked among all classes, and the general sympathy for the Royal Families of both England and Germany, is mingled thankfulness that her Imperial Majesty has been released from sufferings which had often been continuous. Though borne with fortitude, and indeed without complaint, it is no secret that at times they were almost more than she could endure. The end came swiftly—so swiftly that the German Emperor was only just in time to say farewell to his mother, and too suddenly to make it possible for the King to reach his sister while she was alive. The nation will share his Majesty's regret that this last satisfaction was denied to him. There will always be differences of opinion as to the part the Empress, a woman of remarkable mental power and high courage, played in the great world of politics; but there are none as to the exceeding nobility of her private life. In all works of mercy, in all efforts to promote the well-being of society in the Fatherland, and especially in every movement for the benefit of her sex, she was foremost, finding time and opportunity, while not neglecting her duties as wife, mother, and Empress, to fulfil as far as possible the high ideals she had formed as the result of her splendid training and the magnificent example always before her. If she had not been in a Royal position she might easily have become a very successful artist. At fifteen she made a drawing called "The Battlefield," which was sold for the benefit of the widows and orphans of the soldiers who fell in the Crimea, and fetched 500 guineas. In the last week of her life she was engaged in sketching a pedestal for her husband's monument.

---

Countless stories illustrating the gracious manner and kindness of heart of the Empress are told and will be told. Yet, perhaps, nothing more characteristic can be cited than the account of her reception of one of the numerous deputations of school children who went to inquire about the health of her husband when he was ill. One day more than forty little girls of a public elementary school, none of them more than nine or ten years old, attired in their best clothes and escorted by their teacher, arrived at Charlottenburg to give the Emperor the pleasure of receiving some flowers they had bought for him with their scanty pocket-money. They consisted of some fresh and fragrant lilies of the valley, his favourite flower, arranged in a tasteful little basket, which the children presented to the Empress outside the palace. Her Majesty then invited the master and three of the girls inside, handed the gift to a lackey for the Emperor, and disappeared with him for a little while. On her return she told the master and his deputation that she had given the flowers to the Emperor, "who had been extremely touched and pleased at the offering, and had requested her to thank the little donors most cordially for their beautiful present." This message, as may be imagined, caused immense enthusiasm amongst the children. The Empress Frederick then talked to the master for a little while on educational matters, and finally very graciously dismissed him and his charges. Even in the midst of her anxiety about her beloved consort, her Majesty would not neglect the interests, nor spoil the pleasure, of others. There was nothing of self-absorption about her. From first to last she recognised to the full extent the responsibilities of her exalted station, and discharged them, not only conscientiously, but with care that her mode of performing them should be appreciated. The whole world is the poorer for her departure.

---

THOSE who travelled in Norway a good many years ago have been heard to remark that the Norwegian folks were so honest that it would be quite safe for a man who found himself too hot to hang his coat over the hedge on his outward journey, knowing that he could pick it up as he returned. Judging by an adventure which befell the German Emperor when he was there lately, even the influx of foreigners does not appear to have spoilt the simple nature of the Norwegian peasantry. Proceeding from Gudwangen to Stalheim, the Kaiser drove himself in a carriole. Apparently he did this without gloves, and when the groom was cleaning the chaise after he found a ring at the bottom. His Majesty had evidently not discovered his loss, but the man took the treasure trove to the hotel keeper, who restored it to its owner. The next day the Royal visitor drove back to Gudwangen, and on alighting presented the coachman with a 50-kroner note as a reward, but the latter refused the gift, saying that not he, but the groom at Stalheim, had found the ring. Struck with the prompt honesty of the man, the Emperor insisted upon his retaining it, and at the same time gave a duplicate for the groom, saying, "I was extremely glad to have got my ring back again, for it was my engagement ring." You probably know that abroad betrothal rings are nearly always exchanged between the happy pair, and that they are valued almost as much as marriage rings.

---

THE "Father of the House of Commons" died at Westminster Hospital on Saturday evening, the victim of an accident caused by an excavation in Parliament Street. This was the designation enjoyed by that fine old English gentleman, Mr. W. W. B. Beach, who had sat in the House from 1857, and was therefore the oldest of its members. He was also one of the most respected, for though he never held office, his irreproachable Parliamentary manner, and his kindness of heart, as well as his ripe experience, earned him the esteem of all parties. He was seventy-five, and but for the accident which ended fatally, might apparently have represented the Andover division of Hampshire for many more years. The fall from the cab which he suffered on Friday night, in consequence of the horse slipping, might have killed any man, It is some consolation to his numerous sorrowing relatives, including his daughter, who was with him till the end, to know that at Westminster Hospital everything that could be effected by medical skill and nursing was done. Curiously enough, his kinsman, Sir Michael Hicks-Beach, now becomes "Father of the House"—a distinction never, I believe, achieved by any statesman holding the high rank of Chancellor of the Exchequer.

---

IT is one thing to concede the reasonable demands of cyclists and automobilists, and another to revolutionise the road system in order to meet their requirements. In his reply to a deputation the other day, Mr. Walter Long, not for the first time, showed that as President of the Local Government Board, he is not inclined to place the interests of a class, however large and important, before the interests of the country. He confessed that he listened with wonder to the proposal that the present roads should be widened or improved, new roads constructed, building estates properly planned so as to provide thoroughfares, additional or alternative, to the main roads, and tramways and light railways constructed in such a way and on such roads as to diminish the congestion of traffic and population. Mr. Long's astonishment probably reached its climax when the deputation calmly suggested that the State should bear half the cost of maintaining main roads, as if the present demands upon the taxpayers were not sufficiently heavy. He, of course, did not deny that special facilities for the new traffic may become necessary, but he intimated that if special provision has to be made for the benefit of cyclists and automobilists, they must expect to pay a proportion of the expense. This points to the possible imposition of a small tax in the future, and if the cost of collection be not a barrier, I do not think that it would be resented by the vast majority of those who appreciate good roads.

### The South African Medal Roll.

THE following is a full list of the nurses who were presented with South African war medals by his Majesty the King on July 27th:—Nursing Sisters L. C. Agg, E. M. C. Anderson, E. J. Atkins, J. Bulman, M. N. M. Brown, E. R. Brown, G. Balfour, L. Basan, A. A. Bowles, E. P. Carruthers, E. J. Cash, J. Charleson, A. B. Conyngham, F. E. Cross, L. G. Cooley, W. H. Crabtree, J. H. Craw, A. E. Cable, T. A. Cobbold, E. S. Cooper, B. A. Cox-Davies, E. J. Cumming, F. Colborne, A. E. Davidson, A. F. Draper, G. Digwood, G. E. Francis, A. Fisher, E. W. Flanders, M. C. Fisher, G. Fletcher, A. A. Flowers, C. Fullager, M. J. Gordon, M. W. Goodhue, M. A. C. Grant, A. Grantham, C. Hanbury, E. M. J. Halkett, R. G. Hill, A. Hilson, R. M. Hoare, E. M. Howard, B. Huston, C. M. Harvest, E. M. Herriott, A. F. Hislop, B. J. Jones, W. G. Jones, A. E. Joscelyne, J. S. Lambe, S. Lees, M. T. Lightfoot, M. Lowe, F. B. Lambert, E. A. Lee, E. Lewis, M. L. Loveday, M. A. Makins, A. J. McCulloch, M. Miller, G. E. Morgan, E. McCaul, A. Nixon, M. F. Pick, M. Penrose, H. Richardson, N. Redstone, A. Richardson, E. Richardson, A. G. Rogers, G. Ronaldson, M. Rogers, H. Rooke, M. E. Rowell, E. C. Shannon, J. R. Stenhouse, J. E. Skillman, M. J. Spencer, C. N. E. Steel, M. D. Stephenson, J. Schroder, H. M. Shaw, F. M. Shaw, A. S. Siddons, C. M. Theophilus, N. C. F. Tabuteau, H. F. Thompson, W. D. Tripp, M. F. Truell, A. Tarr, G. Tillott, L. M. Tippetts, H. M. Young, L. A. White, E. A. M. Wilson, E. Wilkin, F. M. Wilkinson, L. J. B. Williams, A. L. Wilson, E. H. Wilson, A. W. Walkinshaw, C. Walker, F. M. G. Warrack, and A. J. Williams.

### Everybody's Opinion.

[Correspondence on all subjects is invited, but we cannot in any way be responsible for the opinions expressed by our correspondents. No communication can be entertained if the name and address of the correspondent is not given, as a guarantee of good faith but not necessarily for publication. or unless one side of the paper only is written on.]

### SUGGESTED REFORMS IN MILITARY NURSING.

"MISS M. L. HAYES, Senior Sister of the Indian Army Nursing Service," writes from Station Hospital, Umballa, Punjab: After reading the article "Suggested Reforms in Military Nursing" in your issue of June 15th, I distinctly think that we of the Indian Army Nursing Service are much better off as far as nursing orderlies go. The regimental plan has drawbacks, particularly in the case of regiments leaving the station and, of course, wishing to take their trained men with them; but, on the other hand, we have none of the drawbacks mentioned by "An Army Sister in South Africa." If after a few weeks we find that an orderly who has come for training is unsuitable for the work (even if he has no vices such as those mentioned in this article) the senior sister merely has to mention the fact, and he is at once returned to regimental duty and another man sent in his place. After some years' experience of all sorts of men I must say that, on the whole, they are a splendid set of men—kind and attentive to their patients, very anxious to learn, and conscientious in carrying out orders.

### NURSING IN WESTERN AUSTRALIA.

"AN AUSTRALIAN NURSE" writes under date of June 26th:—In the NURSING MIRROR of May 18th there is an article on the above subject by "An English Nurse." This lady seems to be very disappointed with our fair land. She has certainly not been in any of our principal hospitals; her experience appears to have been confined to private nursing amongst the middle classes or upon the smaller goldfields. Perth, our principal city, is accommodated with a well-built hospital which, although not very large, is well organised. The staff consists of two resident doctors, a matron, seven sisters and 20 probationers, three orderlies and wardsmaids for each ward. The other towns have larger or smaller hospitals according to the size of the town. There are a number of private hospitals kept by well-trained nurses, who always get the credit of looking well after their patients. Private nurses get from two to three guineas weekly, and I have not yet heard of a trained nurse being asked to do the washing or cooking for the family. "An English Nurse" says it is possibly due to our climate that our patients get on so well, but can this be? Would any climate, however perfect, bring them through a serious illness if they were not treated with care? It is impressed upon a fever case from the day of his admission that he must keep very quiet. Sponging, not bathing in a hip bath, is part of our treatment of typhoid fever; medicines are carefully measured and given at regular times, and we are not quite strangers to "antiseptics." We do our best to carry out our surgical work in a thoroughly aseptic manner. We are represented as a pleasure-loving, easy-going people, but we are not content to let things drift on as they will. No, by the Federal Act we belong to the United States of Australia, and we Westralians want to do what we can to make our nation a great one. I trust that other nurses who know something of nursing in Western Australia will take up the subject, as there is a great deal more to be said about it.

### PRIVATE NURSES' FEES.

"A SYMPATHISER" writes: At the present day there seems a great difficulty in obtaining certificated nurses at fees within the means of the middle classes. Wherever you go you hear the same cry, "Nurses are so expensive." Why is it? I think it is because of the nursing homes or institutions. You cannot get a nurse from one under £1 11s. 6d. per week. That is the lowest fee, and is not inclusive, there being a further fee to be paid to the nurse for washing, etc. In infectious cases the fees are much higher, and when the nurse leaves the case the patient's friends must pay an extra week's fee in order that the nurse may go away for disinfection before taking another case. This would be quite right if isolated apartments were kept by the home or institution for the use of nurses under those conditions, but, on the contrary, the nurse is given a portion of the extra fee and told she may go for a week's holiday. The nurses are in receipt of salaries varying from £25 to £30 per annum. How much of that would a nurse, after clothing herself and paying for her annual holiday, be able to save for her old age? Perhaps £5; and with great care, by the time she reached 60 years of age, she might have saved the sum of £200—a goodly sum to live on for the rest of her life! In most "homes," if not in all, the nurses are urged to sign—soon after being engaged, not before—an agreement, binding them not to set up nursing on their own account within 10 miles, until after the expiration of five years; practically the whole of London is debarred them. A nurse belonging to an institution brings into it about £80 per annum, and receives £30 at the most. Where does the rest go? However hard the nurse works, whatever risks she runs, however patient and gentle she may be, no personal benefit accrues to her. If she is disengaged it is true she lives in "The Home," but on all hands I hear the same cry—that the diet is meagre in the extreme, and that the comforts are nil. The restrictions are also very great. A nurse's duties are very arduous, and if she is to be fit mentally and physically, she must be sufficiently and properly fed, and have comfort when off duty. If the nurses would combine and the doctors support them, I think that patients would be able to get certificated nurses at less expense, while at the same time the nurses would gain more and have greater independence. Doctors would have better nurses, inasmuch as there would be greater inducement to the nurses to do their utmost to create a good reputation for themselves. Nurses would have more cases because their lower fees would bring them within the range of the immense middle-class. But how to combine? I suggest "Nurses' Club Homes" in various districts, on a self-supporting basis after the first outlay on furnishing; the expenses to be met by annual subscriptions. The nurses when disengaged and using the Club Home to pay for their board at a little more than cost price. The house rent and salary of a working housekeeper to be met by the annual subscription. The scheme which I propose is that six or seven nurses, or more, should rent a small house just large enough for themselves and a working-housekeeper, and each pay a subscription of £10 per annum (or only the rent of one small room at 4s. a week), which would be sufficient to pay rent and taxes, and the housekeeper's salary. The slight profit on cost price of board would nearly defray the cost of the housekeeper's board. At least a nurse, unless very unfortunate, would have as much money in addition to her own home as if she were in an institute, and would have her liberty for rest or recreation when disengaged. If she made herself of value to doctors and patients she would always be in demand, and it would be a bad year when she could not earn, over and above the subscription, £40 for herself.

### A NURSE'S SUGGESTION TO PUBLIC BODIES.

"A. M. A." writes: When the Local Government Board district councils, or other public bodies having charge of rural districts are called upon to provide nurses for small epidemics of infectious diseases, would it not be found possible, at least in the majority of cases, to put all the cases together, either in a cottage, mission room, corrugated iron temporary hospital, or even in tents? If this could be done the results would be so much more satisfactory, the work lighter, and the patients and nurses would be saved much unnecessary suffering which at present is unavoidable when the patients are treated in their own homes, and the nurses have to go from house to house to attend to them, often walking many miles a day, in all weathers. I was nursing some time ago, in a village in the Midlands, where there was an epidemic of typhoid fever. I arrived about 9 o'clock one night in the autumn, thoroughly chilled, having driven five miles from the station in an open trap, and was taken to a cottage in a lonely spot on the edge of a common to relieve another nurse, who had been sitting up for three nights and days, with a mother and two small children, aged six and two years. The poor nurse looked worn out, and I was glad to be there to take her place. On looking at the former I saw that her suffering was only a question of hours. The eldest child lay on a chair bedstead and was delirious, and the other small child, who was in a cot close by, was in a high state of fever and calling incessantly for its mother. At 3 A.M. the poor woman died, and as there was no other room into which I could move the sick children, the living and the dead had to remain in the same chamber till the next day, when the village carpenter brought the coffin and removed the body to a room downstairs. This was one of the most trying experiences which I have ever had. In the morning the father—a gardener, and a man who drank heavily, as I afterwards found out—asked me to go into the second bedroom and tell his other children of their loss, when I found, to my horror, that in addition to my two small patients, five other children had been made motherless during the night. In the sad circumstances the other nurse and I found it impossible to leave the two sick children night or day with no one but the eldest girl, a child of thirteen, to look after them; yet, in addition to this one cottage, where we practically lived, scrubbing the floor, doing washing, cooking, etc., we had eight other patients to visit, who lived long distances from each other. Between our visits we had to leave the patients to be looked after by their relatives, who almost invariably failed to carry out our instructions. This was essentially the case with regard to the burning of stools, which they looked upon as quite unnecessary and only one of "Nurse's fads." Their disobedience, of course, vastly increased the chances of the infection spreading. One woman was so obstinate on the subject that the sanitary inspector had to threaten her with the police—a policy which only resulted in her resorting to subterfuge, she pretended that she had burnt the stools when all the time she had only emptied them into an outside closet, which was shared by a young married couple living in the next cottage. It was not until the young husband contracted the fever and died that this woman's duplicity was discovered. In another cottage a woman had nursed her husband and little boy with our assistance, but getting very little sleep and rest, and being underfed, she soon contracted the disease herself.

This would not have happened had the cases been removed, as I advocate, to a centre of some sort. Now our great difficulties began. The other people in the village had become panic-stricken as first one person and then another was taken ill, and we found it impossible to get a charwoman or anyone to go in and look after this poor family while we were paying our other visits; and so, after giving them their feeds, we had to leave them for two hours at a time alone, and then return to clean up the house, break coal, chop wood, etc. In this way we managed to get through; but it was a herculean task, and most unsatisfactory to a real nurse to be able to do so little for her patients when so much attention was necessary. If these patients had all been together in one building, the work would have been comparatively light, and the patients would have been more comfortable and had fewer relapses; also the nurses would have been less fatigued and have done their work more cheerfully and with lighter hearts. Never shall I forget the long tramps on dark nights through the rain and fog, from one end of the village to the other, with only a smoking lantern to guide us. Added to this we had no home comforts and often only bare boards to sleep upon. These we were careful to scrub with disinfectant to keep off the fleas! So little comfort and such unsatisfactory results makes a nurse's life a very hard one on these occasions, but with a little thought and management much of it might so easily be avoided.

### Appointments.

GREEK HOSPITAL, ALEXANDRIA.—Miss Florence E. Jones has been appointed sister of the male wards of the Greek Hospital, Alexandria, Egypt. She was trained at the Warneford and South Warwickshire General Hospital, Leamington. Miss Alice E. Swain has been appointed sister of medical and surgical sections, for the women and children's wards of the same hospital.

HUDDERSFIELD SANATORIUM.—Miss L. Hammond has been appointed sister. She was trained at the Union Infirmary, Leeds, and at the City Hospital, Birmingham, and has since been charge nurse at the Union Infirmary, Leeds, and nurse at the Wolverhampton Borough Hospital.

PARK FEVER HOSPITAL, HITHER GREEN.—Miss Elizabeth G. Hughes, Miss A. L. Burgwin, Miss C. Sutton, and Miss A. E. Storey have been appointed charge nurses. Miss Hughes was trained at Sunderland Infirmary, Miss Burgwin at Guy's Hospital, Miss Sutton at Bradford Infirmary, and Miss Storey at Newcastle-on-Tyne Infirmary.

ROYAL ORTHOPÆDIC HOSPITAL.—Miss M. E. Pinsent has been appointed matron. She was trained at the London Hospital, and has been senior sister at the Royal Orthopædic Hospital for the past eighteen months.

WALSALL AND DISTRICT HOSPITAL.—Miss L. L. Rose has been appointed charge nurse. She was trained at the Walsall and District Hospital. She has since been staff nurse and sister at the Guest Hospital, Dudley—with charge of the theatre—and sister of a surgical ward at Wolverhampton General Hospital.

### For Reading to the Sick.

### THE ETERNAL HAVEN OF THE SOUL.

SUBTLEST thought shall fail and learning falter,
Churches change, forms perish, systems go,
But our human needs, they will not alter,
Christ no after age shall e'er outgrow.

Yea, amen! O changeless One, Thou only
Art life's guide and spiritual goal,
Show the Light across the dark vale lonely—
Thou, the Eternal Haven of the soul.—*J. C. Shairp.*

. . . A little while,
And all things shall be new; the night of earth
Shall pass away for ever; "no more sea"
Shall then be found; for pain and loss and grief
Are swallowed up in radiant victory.
Yet, in the country of eternal spring,
Many shall bend to kiss the Master's feet,
Saying, "He never smiled so sweet before,
Save on the sea of sorrow, when the night

Was saddest on our heart. We followed him
At other times in sunshine. Summer days
And moonlight nights He led us over paths
Bordered with pleasant flowers: but when His steps
Were on the mighty waters—when we went
With trembling hearts through nights of pain and loss—
His smile was sweeter and His love more dear;
And only Heaven is better, than to walk
With Christ at midnight over moonless seas."

*B. M.*

God's message to every sad and desolate heart on earth is that God is Light, and in Him is no darkness at all; that God is Love, and in Him there is no cruelty at all; that God is One, and in Him there is no change at all. And therefore we can pray boldly to Him, and ask Him to deliver us in the time of our tribulation and misery; in the hour of death, whether of our own death or the death of those we love; in the Day of Judgment, whereof it is written, "It is God that justifieth us; who is he that condemneth? It is Christ that died, yea, rather, that is risen again, who also maketh intercession for us."—*Charles Kingsley.*

God's calls do not lead men away from their ordinary duties except when He opens the way very clearly for them to embrace some more definite work for Him. God wills us to live for Him in the path of everyday life, and it is there that His special calls come to us. In order to hear the Voice of God we need not turn away from the ordinary circumstances of our life, for it is a uniform law of God's dealings with men that He speaks to them, not at moments when an excited religious fancy would lead them to expect it, but amidst the daily duties of that sphere in which He has placed them.—*G. Body.*

Beware of taking too narrow a view of what is meant by *the world.* It is not confined to any actual intercourse with society. We carry it about us; into our retirements, our houses, our churches. Being in us, in fastens on spiritual things, and will manifest itself through them as an inner spirit. It comes out in the love of rule, in self-pleasing, and in self-complacency, in jealousies, in desire of praise and love of notoriety, and esteem from others. It may thus transform outward piety into what is only another shape of worldliness.—*R. W. Sibthorp.*

### Notes and Queries.

**The Editor is always willing to answer in this column, without any fee, all reasonable questions, as soon as possible.**
**But the following rules must be carefully observed:—**
**1. Every communication must be accompanied by the name and address of the writer.**
**2. The question must always bear upon nursing, directly or indirectly.**
**If an answer is required by letter a fee of half-a-crown must be enclosed with the note containing the inquiry.**

*Case Book.*

(182) I have had printed a case book for private nurses from my own design; will you kindly tell me how I can have it entered at Stationers' Hall; and also if it would be an advantage to have the copyright?—*L. S.*

Your publisher will attend to these things if you give him instructions. The copyright is, of course, yours, until you sell or transfer it.

*Memory.*

(183) Is there any book that would serve to help a nurse to remember what is required for private operations, including gynæcological, and the subsequent treatments?—*Medico.*

"On Preparation for Operations in Private Houses," by Stanmore Bishop, F.R.C.S., price 6d. from the Scientific Press would be useful.

*Bonus.*

(184) Can any nurse who is receiving a pension of £20 a year from the Royal National Pension Fund for Nurses tell me by what sum the added bonus increases it per year?—*G. O.*

The bonus is the profit earned by the Fund during the year. Therefore, it is impossible to tell how much it will amount to before the returns for any particular year are made. The bonuses of this Fund are, as a rule, very good.

*Maternity Books.*

(185) Can anyone tell me what books I ought to study in preparing for the L.O.S. certificate?—*R. H.*

Your teacher will recommend the text books he prefers, but you will probably find a "Practical Handbook of Midwifery," by Francis W. N. Haultain, M.D., price 6s., useful.

*Adoption.*

(186) Will anyone tell me where I can obtain information respecting hospitals and homes for the adoption of children from birth?—*L. G.*

Write to the Association for placing Orphans in Private Families, 98 Cheyne Walk, S.W.; The Foundling Hospital, Guildford Street, Russell Square, W.C.; Dr. Barnardo's Homes, 18 to 26 Stepney Causeway, E.; and The Church of England Society for Waifs and Strays, Savoy Street, Victoria Embankment, W.C.

*Mental Nurse.*

(187) 1. Is there any registry or association for helping mental nurses? I have had seven years' experience in a county asylum, and possess a good character, yet I find it difficult to obtain employment. 2. What class of patients are treated on the Weir-Mitchell system; why is it so called; and, if it is a good calling for women, where can I be trained?—*Stranger.*

1. The Hon. Secretary, the Association of Asylum Workers, Ancaster House, Richmond, Surrey, would probably give you information and advice. 2. The Weir-Mitchell treatment is named after the inaugurator of the system; it is for neurotic patients, and nurses can acquire the necessary instruction in various private establishments.

*Army Nursing Reserve.*

(188) Will you kindly tell me if I ought to have sent the original copies of my certificate and testimonials when I applied to be enrolled in the Army Nursing Reserve?—*Nurse W.*

First write for an official "form of application" and then follow the directions there given.

*Unpaid Fees.*

(189) Will you kindly tell me the best way of collecting my unpaid fees? I am a maternity nurse and three of my patients owe me part of my fee, and refuse to pay it, although they can well afford to do so.

*Q. C. and L. O. S.*

You might put the matter into the hands of a debt collector, or consult a solicitor.

*Convalescent Home.*

(190) Could you kindly give me the address of a convalescent home where a man recovering from diphtheria could be received for about six weeks? He can afford to pay a small sum.—*H. S.*

There are a number of such homes for which see "Burdett's Hospitals and Charities." Perhaps the Merchant Taylors' Convalescent Home for Men, at Bognor, might suit. Apply, the Secretary, The Hall, Threadneedle Street, E.C., but much depends upon the extent to which the patient is free from infection.

*Height.*

(191) What is the regulation height, if any, for members of the Army Nursing Reserve?—*A.B.*

There is no official regulation as to stature.

**Standard Books of Reference.**

"The Nursing Profession: How and Where to Train." 2s. net; post free 2s. 4d.

"Burdett's Official Nursing Directory." 3s. net.; post free, 3s. 4d.

"Burdett's Hospitals and Charities." 5s.

"The Nurses' Dictionary of Medical Terms." 2s.

"Burdett's Series of Nursing Text-Books." 1s. each.

"A Handbook for Nurses." (Illustrated.) 5s.

"Nursing: Its Theory and Practice." New Edition. 3s. 6d.

"Helps in Sickness and to Health." Fifteenth Thousand. 5s.

"The Physiological Feeding of Infants." 1s.

"The Physiological Nursery Chart." 1s.; post free, 1s. 3d.

"Hospital Expenditure: The Commissariat." 2s. 6d.

All these are published by THE SCIENTIFIC PRESS, Ltd., and may be obtained through any bookseller or direct from the publishers, 28 and 29 Southampton Street, London, W.C.

### Travel Notes.

By Our Travelling Correspondent.

### LXXVIII.—THE MUSÉE PLANTIN AND MAISON HYDRAULIQUE.

It will take you at least three hours to see the Musée Plantin, which is, I think after the Cathedral, the most interesting sight in Antwerp. It has only been open to the public during the last twenty-six years, when the family sold it to Government, and the building with all its appurtenances became the property of the city of Antwerp. It is in the little square called the Marché du Vendredi, and was originally the home of Christopher Plantin. He was a celebrated printer, and he set up his press here in 1549, subsequently he was joined by his son-in-law Moretus, and so the firm became that of Plantin and Moretus. They were essentially learned men, and set up works in all languages, and even in Oriental types. In 1579 Philip II. appointed them Royal Printers, and granted them the monopoly for printing all the Mass books, but at the end of the seventeenth century this privilege was withdrawn, and the commercial activity of the Plantin-Moretus firm began to wane. The great interest of the building lies in the fact that nothing has been altered either in the house, the offices, or the shop, and it presents to our gaze a perfect example of the residence and business premises of a wealthy upper-class Fleming in the sixteenth century. I think the shop interested me most; there stands the small counter, the weights and scales and the old ledgers and inkpots. I wish I could give you a full description of the fascinating old building, but it would far outrun my allotted space. There are many splendid portraits of the family and of Justus Lipsius, one of their editors, the latter by Rubens. In the printing offices everything is left arranged as if work was still in progress, the original blocks of their woodcuts and the capital letters are all shown, and in the proof reader's room old proof-sheets still lie on the desks. In the quadrangle an enormous vine hangs all about the walls, clothing them richly with green and yellow; this vine is popularly supposed to have been planted by Christopher himself, and certainly the enormous girth of its stem testifies to it's vast age. In the private part of the building is a magnificent dining hall, of which an artist friend made a most charming picture; the numerous latticed windows are shaded by olive-green faded curtains, and the whole effect is that of subdued richness. There is no sense of solitude; somehow one fully expects the family to appear suddenly and take up once more the threads of life. Over the centre of the long table hangs a truly magnificent Venetian chandelier. On what bright beautiful faces that fairy light must have shone, and to what intellectual and literary conversation it must have lent its twinkling eardrops!

### The Maison Hydraulique.

No guide-book mentions this most interesting building, which, in its own line, is quite as striking as the Plantin house. I should have known nothing of it but for the kindness of a young artist staying in the hotel, who had unearthed this treasure of the past. It is near to the Canal aux Charbons, but hides itself so successfully from the enterprising explorer that it is only found after much search. Moreover, in that part of Antwerp French is little understood, but we were furnished with two Flemish words, "Brouwer" and "Haus," and by diligent repetition of these to many grinning, but most obliging, natives we finally achieved our goal. The Brewery is managed exactly as it was 200 years ago, and with the same machinery. The latter was all turned by a horse who tramped round and round in an underground stable in the way one sometimes sees in an English farmyard. Somewhat below this was a swinging bridge by which we crossed a weird and awesome black canal. It was really a most singular and uncanny place. I asked permission to sketch here, which was readily granted, and I returned to the hotel for my traps.

### An Unexpected Catastrophe.

I established myself in the lightest place, namely, on some steep steps which led up to the edge of the Canal aux Charbons, from which I looked down on a species of subterranean cellar, below which again was the wicked-looking water crossed by the frail bridge. I had, fortunately, nearly finished a hasty sketch when, with horrid unexpectedness, a perfect deluge of water swooped upon me from above, rushing down the steps and literally lifting me from my perch. It was a large water cart which, when filled, had been turned round suddenly and launched a heavy body of water upon the votary of art seated below at the bottom of the shaft. It is no exaggeration to say I had not a dry thread on me; there was nothing to be done but to return to the hotel and finish the beauties of the Maison Hydraulique another day, which we did. Our artist friend was painting in the large banqueting room reserved for the Guild of the Brewers, a magnificent room hung with Spanish leather. Just outside it was a charming little oaken stair with a carved figure standing on the last thick banister. I should much like to have secured photographs of this interesting building, but there was no such thing to be had, and I was forced to content myself with my hasty sketches secured under such unfavourable circumstances. There is not much costume to be seen now either in Holland or Belgium. Some of the women cannot quite make up their minds to part with the exquisite Flemish lace caps with long lappets and the Dutch golden skull caps with twisted horns on each temple, both these adornments being heirlooms, so they compromise matters and meet the difficulty by surmounting the whole structure with a modern bonnet adorned with artificial flowers! The chief photographer in the town, a most intelligent and artistic man, showed us a portrait of one of these up-to-date ladies, and groaned out with a most pained expression on his face, "Mais Madame c'est affreuse!"

---

### TRAVEL NOTES AND QUERIES.

Meals in Germany (Tarascon).—Yes, the dinner hour is usually 1 o'clock and supper at 7 or 7.30, but under your circumstances I think in many places they would be willing to meet your requirements. You can understand, however, that as a rule they could not entertain such a proposition, and even if they grant your request, I fear you must be prepared to increase your terms a little. Indeed, that would only be fair. I have found German hotel proprietors most obliging, and generally really kind in helping through any difficulty.

Rome viâ Laon (Mars).—It is a very interesting place and well worth a visit. Usually travellers rush through without a thought of the interest they leave behind. The train from Calais at 2.50 reaches Laon at 7.2. Sleep there the night, you will have all the next day to see the cathedral—built in 1114—the Church of St. Martin, the Prefecture, and the Tower of Louis d'Outremer before taking the train at 7.2, the same hour at which you arrived the night before. If you decide to see Rheims at the same time you can leave Laon early in the day, go on to Rheims and pick up the 7.2 train but that is really too much to do in one day. I should recommend two nights at Laon, visiting Rheims from there to save expense. You certainly want at least three hours at the latter place.

Fribourg or Freiburg (Iota).—Fribourg is in Switzerland, near to Berne—it is the French way of spelling the name. The other Freiburg is in the Black Forest. and is often spelt in the same way. The one you want to see is in the Black Forest. It has a beautiful cathedral with an open-work spire. But Freiburg near Berne possesses a splendid organ in the church of St. Nicholas.

To Belgium (a Countrywoman).—Your best way will be to go by the Tower Bridge route to Ostend, *not* Antwerp, and go straight to Brussels—return, second class, £1 0s. 1d. Whilst at Brussels spend a long day at Antwerp, but don't sleep, it will be considerably cheaper in every way to see it from Brussels. From Brussels you can also see with ease Malines and Louvain. Both these two could be done in one day, that would make three days at Brussels; on the fourth leave by an early train, spend the day at Ghent, but don't sleep, and arrive at Bruges in time for dinner; sleep at that town and leave the next day for the steamer at Ostend. This would make five days instead of three, but the economy effected by not constantly changing trains will make it come to no more. Hotel at Brussels—Hôtel de la Cathédrale, 17 Place de Ste. Gudule, ask for room on *third* floor. Take plain breakfast and table d'hôte there. At Bruges go to Hôtel Panier d'Or in the market-place. Your expenses will come to about 6½ francs a day and 1½ in tips, making for five days, till you go on board, about 40 francs. Write to Messrs. Gaze for your ticket by the steamer and the return to Brussels with extension to Malines and Louvain, and allowing break at Ghent and Bruges. Antwerp must be done separately, I think, but they will tell you. You can pay extra on the boat for 1st class if you think it worth while. A [illegible] when the boats *return* or you might be compelled to remain longer than you wish.

